# Neuronal nitric oxide synthase is required for erythropoietin stimulated erythropoiesis in mice

**DOI:** 10.3389/fcell.2023.1144110

**Published:** 2023-02-21

**Authors:** Jeeyoung Lee, Soumyadeep Dey, Praveen K. Rajvanshi, Randall K. Merling, Ruifeng Teng, Heather M. Rogers, Constance T. Noguchi

**Affiliations:** ^1^ Molecular Medicine Branch, National Institute of Diabetes and Digestive and Kidney Diseases, National Institutes of Health, Bethesda, MD, United States; ^2^ Skeletal Biology Section, National Institute of Dental and Craniofacial Research, National Institutes of Health, Bethesda, MD, United States; ^3^ Laboratory of Pathology, National Cancer Institute, National Institutes of Health, Bethesda, MD, United States

**Keywords:** erythropoietin, nitric oxide, hematopoiesis, erythropoiesis, neuronal nitric oxide synthase, proliferation, transplantation

## Abstract

**Introduction:** Erythropoietin (EPO), produced in the kidney in a hypoxia responsive manner, is required for red blood cell production. In non-erythroid tissue, EPO increases endothelial cell production of nitric oxide (NO) and endothelial nitric oxide synthase (eNOS) that regulates vascular tone to improve oxygen delivery. This contributes to EPO cardioprotective activity in mouse models. Nitric oxide treatment in mice shifts hematopoiesis toward the erythroid lineage, increases red blood cell production and total hemoglobin. In erythroid cells, nitric oxide can also be generated by hydroxyurea metabolism that may contribute to hydroxyurea induction of fetal hemoglobin. We find that during erythroid differentiation, EPO induces neuronal nitric oxide synthase (nNOS) and that neuronal nitric oxide synthase is required for normal erythropoietic response.

**Methods:** Wild type (WT) mice and mice with targeted deletion of nNOS (*nNOS−/−*) and eNOS (*eNOS−/−*) were assessed for EPO stimulated erythropoietic response. Bone marrow erythropoietic activity was assessed in culture by EPO dependent erythroid colony assay and in vivo by bone marrow transplantation into recipient WT mice. Contribution of nNOS to EPO stimulated cell proliferation was assessed in EPO dependent erythroid cells and in primary human erythroid progenitor cell cultures.

**Results:** EPO treatment increased hematocrit similarly in WT and *eNOS−/−* mice and showed a lower increase in hematocrit *nNOS−/−* mice. Erythroid colony assays from bone marrow cells were comparable in number from wild type, *eNOS−/−* and *nNOS−/−* mice at low EPO concentration. Colony number increased at high EPO concentration is seen only in cultures from bone marrow cells of wild type and *eNOS−/−* mice but not from *nNOS−/−* mice. Colony size with high EPO treatment also exhibited a marked increase in erythroid cultures from wild type and *eNOS−/−* mice but not from* nNOS−/−* mice. Bone marrow transplant from *nNOS−/−* mice into immunodeficient mice showed engraftment at comparable levels to WT bone marrow transplant. With EPO treatment, the increase in hematocrit was blunted in recipient mice that received with *nNOS−/−* donor marrow compared with recipient mice that received WT donor marrow. In erythroid cell cultures, addition of nNOS inhibitor resulted in decreased EPO dependent proliferation mediated in part by decreased EPO receptor expression, and decreased proliferation of hemin induced differentiating erythroid cells.

**Discussion:** EPO treatment in mice and in corresponding cultures of bone marrow erythropoiesis suggest an intrinsic defect in erythropoietic response of *nNOS−/−* mice to high EPO stimulation. Transplantation of bone marrow from donor WT or *nNOS−/−* mice into recipient WT mice showed that EPO treatment post-transplant recapitulated the response of donor mice. Culture studies suggest nNOS regulation of EPO dependent erythroid cell proliferation, expression of EPO receptor and cell cycle associated genes, and AKT activation. These data provide evidence that nitric oxide modulates EPO dose dependent erythropoietic response.

## 1 Introduction

Erythropoietin (EPO), a hormone produced in the kidney in a hypoxia inducible manner, is the principle regulator of red blood cell production for transport of oxygen from the lungs to the tissues (Bunn, 2013). EPO acts by binding to its cell surface EPO receptor (EPOR) on erythroid progenitor cells to promote survival, proliferation, and differentiation to produce mature erythrocytes (Bhoopalan et al., 2020). Loss of *EPO* or *EPOR* causes death *in utero* due to severe anemia (Wu et al., 1995; Lin et al., 1996). EPOR is also expressed in non-erythroid tissues contributing to EPO activity beyond red blood cell production (Suresh et al., 2019). Bioavailability of nitric oxide (NO) in the vasculature contributes to regulation of local blood flow affecting oxygen delivery. EPOR expression in endothelial cells mediates EPO stimulated activation of endothelial nitric oxide synthase (eNOS) and endothelial production of NO (Beleslin-Cokic et al., 2004; Mihov et al., 2009a; Premont et al., 2020). EPO stimulated increase in endothelial eNOS activation and NO production were required for EPO cardioprotection in an acute mouse model of ischemia-reperfusion injury (Mihov et al., 2009a; Teng et al., 2011). EPO also promotes angiogenesis and an increase in intracellular calcium mediated in part by transient receptor potential vanilloid type 1 (TRPV1) and required phospholipase C-gamma1 activity (Xue et al., 2011; Yu et al., 2017). Absence of EPO signaling during embryogenesis results in angiogenic defects (Kertesz et al., 2004; Watanabe et al., 2005; Nakano et al., 2007). In a mouse myocardial ischemia/reperfusion model, EPO can promote nNOS expression contributing to angiogenesis and protection against ventricular arrhythmia (Burger et al., 2009; Wen et al., 2014). The presence of eNOS in erythrocytes and the potential for EPO to activate eNOS and NO production in red blood cells further advances EPO regulation of NO bioavailability and vascular tone (Kleinbongard et al., 2006; Mihov et al., 2009b; Simmonds et al., 2014).

NO and NOS may directly affect EPO stimulated erythropoiesis. NO treatment in wild type mice shifted hematopoiesis toward the erythroid lineage and reduced leukocyte counts, mediated in part by activation of soluble guanylate cyclase (Ikuta et al., 2016). A role for NO to modify the erythroid program was suggested by hydroxyurea induction of fetal hemoglobin mediated by NO-dependent activation of soluble guanylyl cyclase (Cokic et al., 2003). In patients with end stage renal disease, levels of erythrocyte asymmetric dimethylarginine, a naturally occurring inhibitor of NOS, was associated with low hemoglobin levels and erythropoietin resistance (Yokoro et al., 2017). Similarly, a mouse model for advanced chronic kidney disease exhibited decreased hemoglobin, hematocrit and splenic *EPOR* gene expression as well as increased erythrocyte asymmetric dimethylarginine, suggesting that erythrocyte accumulation of asymmetric dimethylarginine and suppression of *EPOR* contribute to impaired erythropoietin response (Yokoro et al., 2017).

In the current study, NOS requirement for EPO stimulated erythropoiesis was determined by EPO treatment in mice with deletion of *eNOS* (*eNOS−/−*) or *nNOS* (*nNOS−/−*). *nNOS−/−* mice showed a blunted erythropoietic response compared with wild type (WT) mice. Cultures of hematopoietic progenitor cells from *eNOS−/−* and *nNOS−/−* mice treated with EPO confirmed the requirement of nNOS for erythroid colony formation, especially at high EPO concentration. Bone marrow transplantation provided additional evidence for nNOS activity in hematopoietic progenitor cells for normal EPO dependent erythropoietic response. Treatment of erythroid cell cultures indicated that nNOS inhibition decreased EPOR expression and EPO dependent erythroid cell proliferation mediated in part by altered cell cycle gene expression and decreased AKT activation.

## 2 Materials and methods

### 2.1 Animals

Animal procedures were approved by the National Institute of Diabetes and Digestive and Kidney Diseases Animal Care and Use Committee and carried out in accordance with the National Institutes of Health Guidelines for the Care and Use of Laboratory Animals. Mice were maintained under a specific pathogen–free and thermostable environment (23°C) and photoperiod conditions (12/12 h light/dark cycle) with free access to food (NIH-31, 14% kcal/fat, 3.0 kcal/g, Teklad Diets) and water. All mice were on a C57BL/6 background. *eNOS−/−* mice and *nNOS−/−* mice were purchased from Jackson Laboratory (stock no. 002684 and 002986, respectively). *nNOS−/−* mice were bred by mating heterozygous females to homozygous males or by mating homozygous females to heterozygous males. Mouse genotype was determined at weaning by PCR analysis of extracted DNA. Pep Boy with CD45.2 mice (Jackson Laboratory, stock no. 002014) were used as recipient mice for bone marrow transplantation.

### 2.2 EPO treatment and hematocrit measurements

Mice (10–12 weeks old) were treated with EPO (recombinant human EPO, Epogen, Amgen, Thousand Oaks, CA, United States) at 3,000 units/kg three times/week for 7–10 days as indicated. EPO was administrated by intraperitoneal injection. For hematocrit, blood was collected from the tail vein in heparin coated capillary tubes. The tubes were centrifuged using a micro-hematocrit centrifuge (Unico, NJ, United States) and hematocrits were measured using a VIN micro-hematocrit capillary tube reader (Veterinary Information Network Bookstore, CA, United States).

### 2.3 Colony formation assay

125,000 *de novo* isolated cells from mouse bone marrow were plated in MethoCult M3334 (Stem Cell Technologies, Vancouver, Canada) containing SCF, IL-3, IL-6 (PeproTech, Rocky Hill, NJ, United States), and EPO (Amgen, Thousand Oaks, CA, United States) at indicated doses, and cultured at 37°C and 5% CO_2_. Colonies were counted and scored at 14 days post-plating using phase contrast microscopy.

### 2.4 Bone marrow transplantation

Recipient female Pep Boy (CD45.2) mice at 8–10 weeks received 20 mg/kg of pharmaceutical grade busulfan by intraperitoneal injection (Hayakawa et al., 2009) 1 day prior to transplantation. Bone marrow cells were collected from female WT and *nNOS−/−* mice at 8–10 weeks and were infused by direct injection intravenously into the tail vein of Pep Boy mice. After 11–12 weeks following transplantation, blood was drawn from the recipient Pep Boy mice by nicking of the tail vein to analyze engraftment rate. Mice were then treated with EPO (Amgen, Thousand Oaks, CA, United States) at 3,000 units/kg three times/week for 1 week and hematocrit levels were determined.

### 2.5 FACS analysis

After erythrocyte lysis, bone marrow cells were incubated with 0.5 µg of anti-mouse CD16/CD32 antibody for blocking Fc receptors. For analyzing hematopoietic cells in the bone marrow, cells were incubated with CD45.1 (#553775, BD Bioscience) and CD 45.2 (#558707, BD Bioscience) for 30 min, followed by washing in staining buffer, and analysis by FACS Calibur (BD Bioscience).

### 2.6 HCD57 and K562 cell culture

EPO-dependent murine HCD57 erythroleukemia cells were grown in Iscove’s modified Dulbecco’s medium (IMDM), 25% fetal bovine serum (FBS), 10 μg/mL gentamicin (Invitrogen, Thermo Fisher Scientific, Grand Island, NY, United States), at 37°C in a 5% CO_2_ environment with either 0.2 U/mL or 2 U/mL EPO (Amgen, Thousand Oaks, CA, United States) (Sawyer and Jacobs-Helber, 2000). The human K562 chronic myeloid leukemia cell line was cultured in RPMI 1640 medium, 10% fetal bovine serum (FBS), and 1% Penicillin/Streptomycin (Invitrogen) at 37°C with 5% CO_2_. To investigate the effect of nNOS inhibitor, HCD57 and K562 cells were cultured with nNOS inhibitor, 7-Nitroindazole (7-NI; #00240 Biotium, Fremont, CA, United States) at different concentrations (10, 100, 200 μM). HCD57 cells were harvested at day 4 for analysis of gene and protein expression. For erythroid differentiation of K562 cells, cells were stimulated with 30 μM of hemin (Millipore Sigma, St. Louis, MO, United States).

### 2.8 Human peripheral blood erythroid progenitor cell cultures

Buffy coat packs were prepared from units of whole blood collected from volunteer donors at the National Institutes of Health, Department of Transfusion Medicine. They were distributed for research use in an anonymized manner, through an exemption from full IRB review granted by the National Institutes of Health Office of Human Subjects Research Protections. Erythroid progenitors were harvested and grown in a two-phase liquid culture system (Fibach, 1998). Mononuclear cells were isolated by centrifugation on Ficoll-Hypaque (1.077 Density, Mediatech, Inc. Corning, Manassas, VA, United States). Cells were then cultured in α-minimal essential medium (αMEM) supplemented with 10% fetal bovine serum (FBS), 1.5 mM L-Glutamine (100 mM), 1% Penicillin/Streptomycin (Invitrogen, Thermo Fisher Scientific, Grand Island, NY, United States), 10% conditioned medium from bladder carcinoma 5,637 cultures, and 1 mg/mL cyclosporin A (Millipore Sigma, St. Louis, MO, United States). Cultures were incubated at 37°C, 5% CO_2_. After 5–7 days, non-adherent cells were washed twice with Dulbecco’s phosphate-buffered saline without Ca^2+^ and Mg^2+^ and transferred to α-minimal essential medium supplemented with 30% fetal bovine serum, 1% Penicillin/Streptomycin (Invitrogen), 1% deionized bovine serum albumin, 10^−6^ M dexamethasone, 10^−5^ M β-mercapthoethanol, 0.3 mg/mL human holo-transferrin (Millipore Sigma), 10 ng/mL human recombinant stem cell factor (PeproTech, Rocky Hill, NJ, United States), and 2 U/mL EPO (Amgen, Thousand Oaks, CA, United States). Cultures were incubated at 37°C, 5% CO_2_ for up to 12 days.

### 2.9 Quantitative real-time RT-PCR

Quantitative real-time RT-PCR analyses were carried out using gene-specific primers (Supplementary Table S1) and fluorescently labeled SYBR Green dye (Roche, Indianapolis, IN, United States) in a 7900 Sequence Detector (Applied Biosystems, Thermo Fisher Scientific, Foster City, CA, United States). For relative mRNA quantification, Ct values were normalized with *RPL13a* as an internal control using the delta-delta CT method.

### 2.10 Western blotting

Cellular proteins were extracted by AllPrep RNA/Protein Kit (Qiagen, Germantown, MD, United States). Isolated proteins were resolved by 4%–20% Tris-glycine SDS/PAGE, transferred to nitrocellulose membranes, blotted using an XCell SureLock Mini-Cell system (Invitrogen, Thermo Fisher Scientific, Grand Island, NY, United States) and visualized using protein specific antibodies (Supplementary Table S2). Quantitative analysis was performed by measuring the integrated density using NIH ImageJ and normalized to GAPDH.

### 2.11 Apoptosis assays

To measure the number of apoptotic cells, annexin V and propidium iodide (PI) staining and flow cytometry were used. After 7-NI treatment for 4 days, HCD57 cells were resuspended in cell staining buffer (#420201, Biolegend) and Allophycocyanin (APC)–labeled and PI (#640932, Biolegend) were added to cells and incubated for 15 min. The number of annexin V+/PI− and annexin V+/PI+ were quantified using FACS Calibur (BD Bioscience).

### 2.12 Statistical analysis

The data are expressed as mean ± s.e.m. Comparisons between two groups were made using Student’s two-tailed non-paired *t*-test. *p* values of <0.05 were regarded as statistically significant.

## 3 Results

### 3.1 EPO treatment in *nNOS−/−* mice results in a blunted erythropoietic response while increased hematocrit in EPO treated *eNOS−/−* mice is comparable to EPO treated WT mice

**FIGURE 1:**
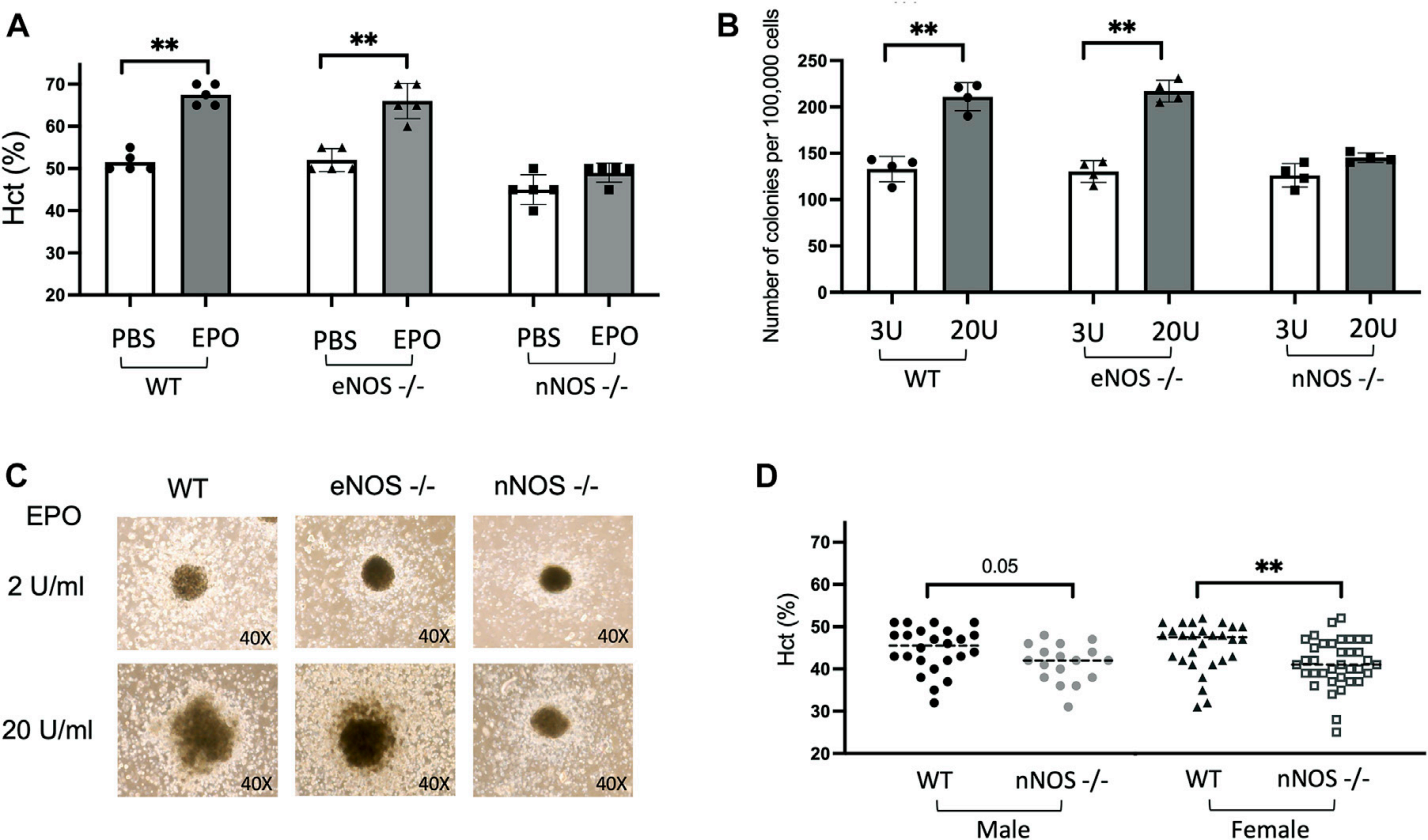
EPO stimulated erythropoiesis in *nNOS−/−* mice. *nNOS−/−* mice showed a blunted erythropoietic response to EPO while erythropoietic response in *eNOS−/−* mice was comparable to WT. **(A)** Hematocrit levels (%) in WT, *eNOS−/−*, and *nNOS−/−* mice were determined after 10 days of EPO treatment (3000 U/kg; 3 times per week). **(B)** Methylcellulose hematopoietic colony-forming assays were carried out using bone marrow hematopoietic cells from WT, *eNOS−/−*, and *nNOS−/−* mice and methylcellulose-based media supplemented with hematopoietic growth factors, EPO at 3 U/mL or 20 U/mL, and scored for total hematopoietic colonies after 14 days. **(C)** Photographs of CFU-granulocyte, erythroid, macrophage, megakaryocyte (CFU-GEMM) colonies derived from bone marrow hematopoietic cells cultured with EPO at 2 U/mL or 20 U/mL from WT, *eNOS−/−* and *nNOS−/−* mice (40X). **(D)** Hematocrit levels (%) in male and female WT and *nNOS−/−* mice (∗*p* < 0.05, and ∗∗*p* < 0.01).

EPO is the primary regulator of erythropoiesis and EPO treatment (3,000 units/kg, three times/week for 10 days) in WT mice beginning at 10 weeks of age stimulates red blood cell production and increases hematocrit from 51.5% ± 1% to 67.5% ± 1.1% (Figure 1A). To examine if nNOS and eNOS contribute to EPO stimulated erythroid response, we also treated 10-week-old *eNOS−/−* and *nNOS−/−* mice with EPO (3,000 units/kg, three times/week for 10 days). EPO treatment in *eNOS−/−* mice resulted in increased hematocrit from 52% ± 1.2% to 66% ± 1.9% (Figure 1A), comparable to EPO stimulated erythropoiesis in WT mice. In contrast, *nNOS−/−* mice showed a blunted erythropoietic response to EPO treatment with only a modest increase in hematocrit from 45% ± 1.6% to 49% ± 1% (Figure 1A). To determine if EPO stimulated erythroid response is differentially regulated in hematopoietic progenitor cells from *nNOS−/−* mice compared with WT and *eNOS−/−* mice, *in vitro* cultures of isolated bone marrow cells from WT, *eNOS−/−* and *nNOS−/−* mice were assessed for erythroid colony formation. At EPO concentration of 2–3 U/mL, erythroid colony formation from bone marrow cells isolated from WT, *eNOS−/−*, *nNOS−/−* mice were similar in number (133 ± 6.8, 130.25 ± 5.9 and 126 ± 6.4, respectively) and in colony size. In contrast, bone marrow cultures treated with higher dose of EPO (20 U/mL) resulted in increased erythroid colony numbers from WT and *eNOS−/−* mice (211 ± 7.5 and 217 ± 5.8, respectively) (Figure 1B) and corresponding increased colony size (Figure 1C), while *nNOS−/−* mice exhibited little or only modest increases in colony number (145 ± 2.5) and size (Figures 1B, C). Without EPO treatment, male *nNOS−/−* mice (*n* = 17) exhibit a trend toward reduced hematocrit compared with male WT mice (*n* = 24) that was significant between female *nNOS−/−* mice (*n* = 36) and female WT mice (*n* = 28) (Figure 1D).

### 3.2 Mice transplanted with *nNOS−/−* donor marrow show reduced response and smaller increase in hematocrit with EPO treatment compared to mice transplanted with WT donor marrow

**FIGURE 2:**
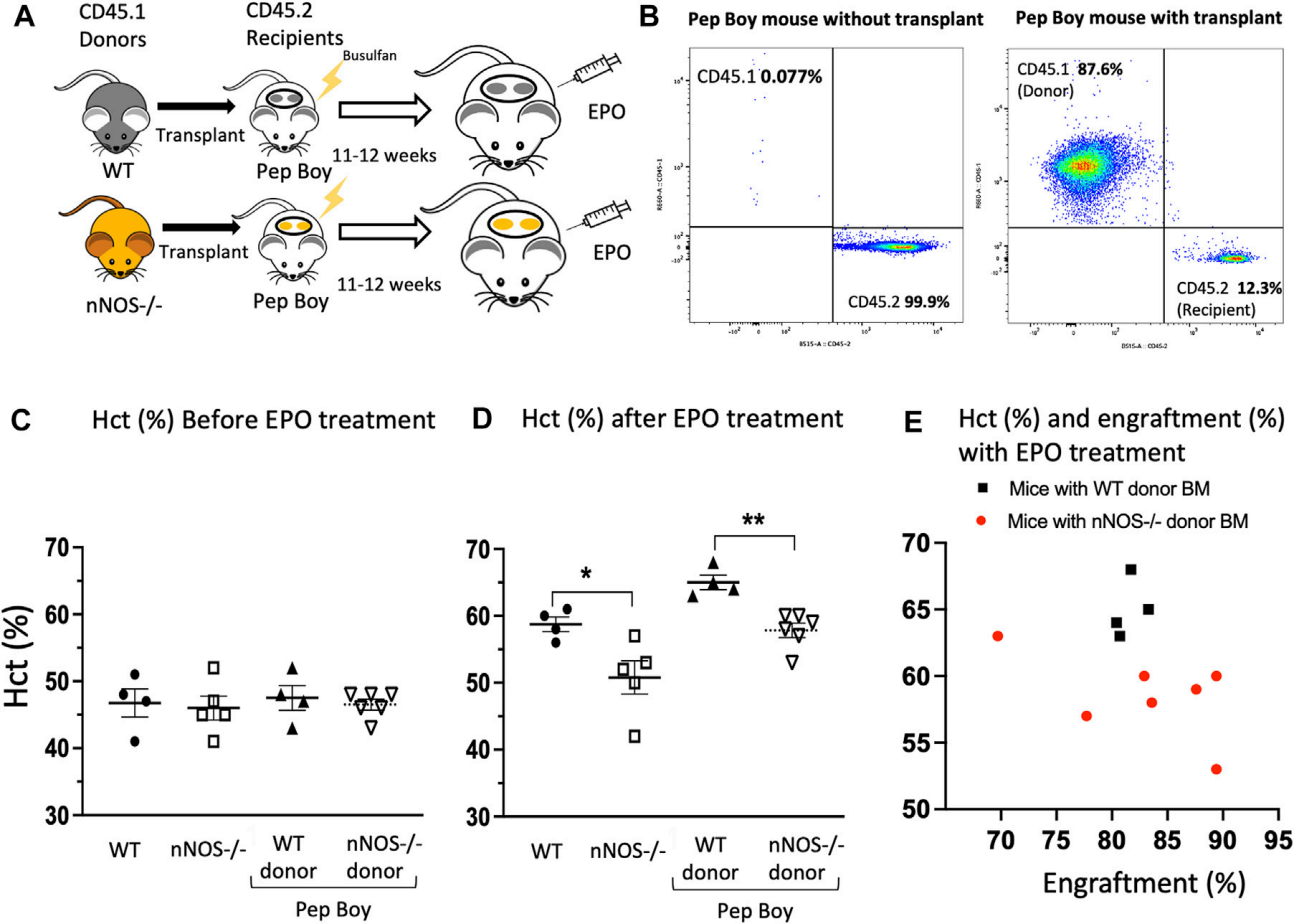
EPO stimulated erythropoiesis in mice transplanted with *nNOS−/−* bone marrow. Mice transplanted with *nNOS−/−* bone marrow exhibited a blunted erythropoietic response to EPO treatment with a smaller increase in hematocrit compared to mice that received WT donor marrow. **(A)** Bone marrow from WT (CD45.1) and *nNOS−/−* (CD45.1) donor mice was transplanted into Pep Boy (CD45.2) recipient mice. **(B)** Representative FACS plots for CD45.1 and CD45.2 cells from peripheral blood of Pep Boy mice with transplant from nNOS−/− mice and without transplant. Engraftment efficiency was measured after 10–11 weeks following transplant. **(C,D)** Hematocrit levels (%) in WT and *nNOS−/−* mice and in Pep Boy recipient mice transplanted with WT and *nNOS−/−* donor marrow were assessed before **(C)** and after **(D)** 1 week of EPO treatment (3000 U/kg; 3 times per week). Only mice with more than 70% transplant rate were analyzed. **(E)** Hematocrit levels (%) were plotted with engraftment (%) for recipient Pep Boy mice with WT (black squares) and *nNOS−/−* (red circles) donor marrow.

Bone marrow transplantation was used to demonstrate that the decreased EPO stimulated erythropoietic response in *nNOS−/−* mice was directly related to a blunted EPO stimulated erythroid response of *nNOS−/−* hematopoietic cells. Bone marrow cells isolated from WT (CD45.1) and *nNOS−/−* (CD45.1) mice were transplanted into Pep Boy mice (CD45.2) after Busulfan conditioning (Figure 2A). At 11–12 weeks posttransplant, transplanted donor marrow cell (CD45.1) from *nNOS−/−* mice (Figure 2B) and WT mice (Supplementary Figure S1) were counted by fluorescence activated cell sorting (FACS) analysis in peripheral blood of recipient Pep Boy mice (CD45.2). This was compared with untransplanted mice. Similar level of engraftment was observed for transplanted bone marrow cells from *nNOS−/−* (81.5% ± 0.7%) and WT mice (82.8% ± 0.7%). Without EPO treatment, hematocrit levels were comparable for all mouse groups, untransplanted WT and *nNOS−/−* mice, transplanted Pep Boy mice with WT donor and transplanted Pep Boy with *nNOS−/−* mice (Figure 2C). Mice were treated with EPO (3,000 U/kg; 3 times per week) for 1 week, 11–12 weeks after transplant. Transplanted mice receiving WT donor marrow showed the greatest increase in hematocrit (65% ± 1.0%) in contrast to the reduced increase in hematocrit with EPO treatment in transplanted mice receiving *nNOS−/−* donor marrow (58.6% ± 1.2%) (Figure 2D). This difference was comparable to hematocrits after EPO treatment in untransplanted WT mice and untransplanted *nNOS−/−* mice, respectively (Figure 2D). The higher hematocrit levels in EPO treated transplanted Pep Boy mice compared with untransplanted mice may reflect a more robust erythropoietic response in Pep Boy mice compared to WT mice. Engraftment of transplanted WT donor bone marrow was consistent between 80% and 83%, while engraftment of *nNOS−/−* donor marrow exhibited a larger range between 70% and 90% and a negative correlation between hematocrit (%) and engraftment (%) (Figure 2E). Mice transplanted with *nNOS−/−* donor marrow that had the lowest level of % engraftment was associated with the highest hematocrit.

### 3.3 nNOS inhibitor decreased cell proliferation of EPO dependent mouse erythroid HCD57 cells and of hemin induced erythroid differentiating human K562 cells

**FIGURE 3:**
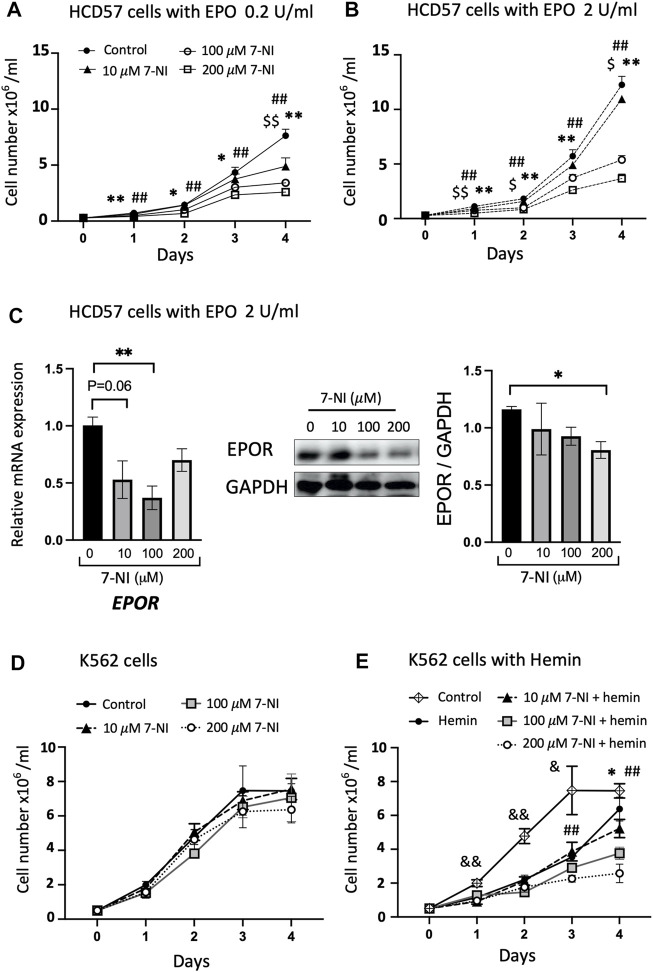
Inhibition of nNOS in proliferating erythroid cells. Treatment with nNOS inhibitor decreases proliferation of EPO-dependent HCD57 cells and proliferation of erythroid differentiating EPO-independent K562 cells. **(A,B)** Growth curves of EPO-dependent HCD57 cells cultured with 0.2 U/mL **(A)** and 2 U/mL **(B)** EPO were determined with addition of increasing concentrations of nNOS inhibitor 7-NI (*n* = 3/group; indicated are $, *, #*p* < 0.05, and $$, **, ##*p* < 0.01; $ means control vs. 10 μM 7-NI, * means control vs. 100 μM 7-NI, # means 200 μM 7-NI). **(C)** *EPOR* gene expression (left panel) and EPOR protein expression determined by western blotting (*n* = 3–4/group) in HCD57 cells were assessed with EPO treatment at 2 U/mL and increasing concentration of nNOS inhibitor. HCD57 cells were harvested at day 4. **(D,E)** Growth curves of EPO-independent K562 cells were determined with addition of nNOS inhibitor cultured without **(D)** and with **(E)** hemin induced erythroid differentiation (*n* = 4/group; indicated are &, *, #*p* < 0.05, and &&, **, ##*p* < 0.01; & means control vs. hemin, * means hemin vs. 100 μM 7-NI + hemin, # means hemin vs. 200 μM 7-NI + hemin).

Cultures of EPO dependent (HCD57) and EPO independent (K562) cells were used to determine if nNOS contributes to erythroid cell growth. Mouse erythroleukemia HCD57 cells are EPO-responsive and require EPO for survival. Human myelogenous leukemia K562 cells are EPO independent and can be chemically induced to undergo erythroid differentiation (Jacobs-Helber et al., 2000). Cultures were treated with increasing amounts of nNOS inhibitor 7-NI (10, 100, 200 μM). HCD57 cells cultured with EPO at 0.2 and 2.0 U/mL exhibited EPO concentration dependent proliferation that was inhibited by increasing concentrations of 7-NI especially at 100 and 200 μM (Figures 3A, B). The treatment with 7-NI at high dose compared with PBS after 4 days of culture in HCD57 cells increased cell death (annexin V + PI+: 7-NI 200 μM at 7.81% *versus* PBS at 4.83%) and apoptosis (annexin V + PI: 7-NI 200 μM at 17.6% *versus* PBS at 16.5%) in flow cytometry (Supplementary Figure S1). However, these changes appear to be small compared with the reduction in cell number by more than 50%. Expression of *EPOR* mRNA and protein showed a decreasing trend in HCD57 cell cultures treated with increasing concentrations of 7-NI (Figure 3C), consistent with the decreased growth response to 7-NI exposure. EPO independent proliferation of K562 cells was not affected by treatment with 7-NI at concentrations from 10 to 200 μM (Figure 3D), suggesting that nNOS may be required for EPO stimulated erythroid cell proliferation, but not for EPO independent erythroid cell growth. Hemin induction of erythroid differentiation of K562 cells decreased cell proliferation. Exposure of hemin induced differentiating K562 cells to 7-NI further decreased cell proliferation (Figure 3E) indicating that nNOS may contribute to maintaining cell growth and erythroid lineage expansion during erythroid differentiation.

### 3.4 Reduced EPO dependent erythroid cell proliferation by nNOS inhibitor is associated with cell cycle gene regulation

**FIGURE 4:**
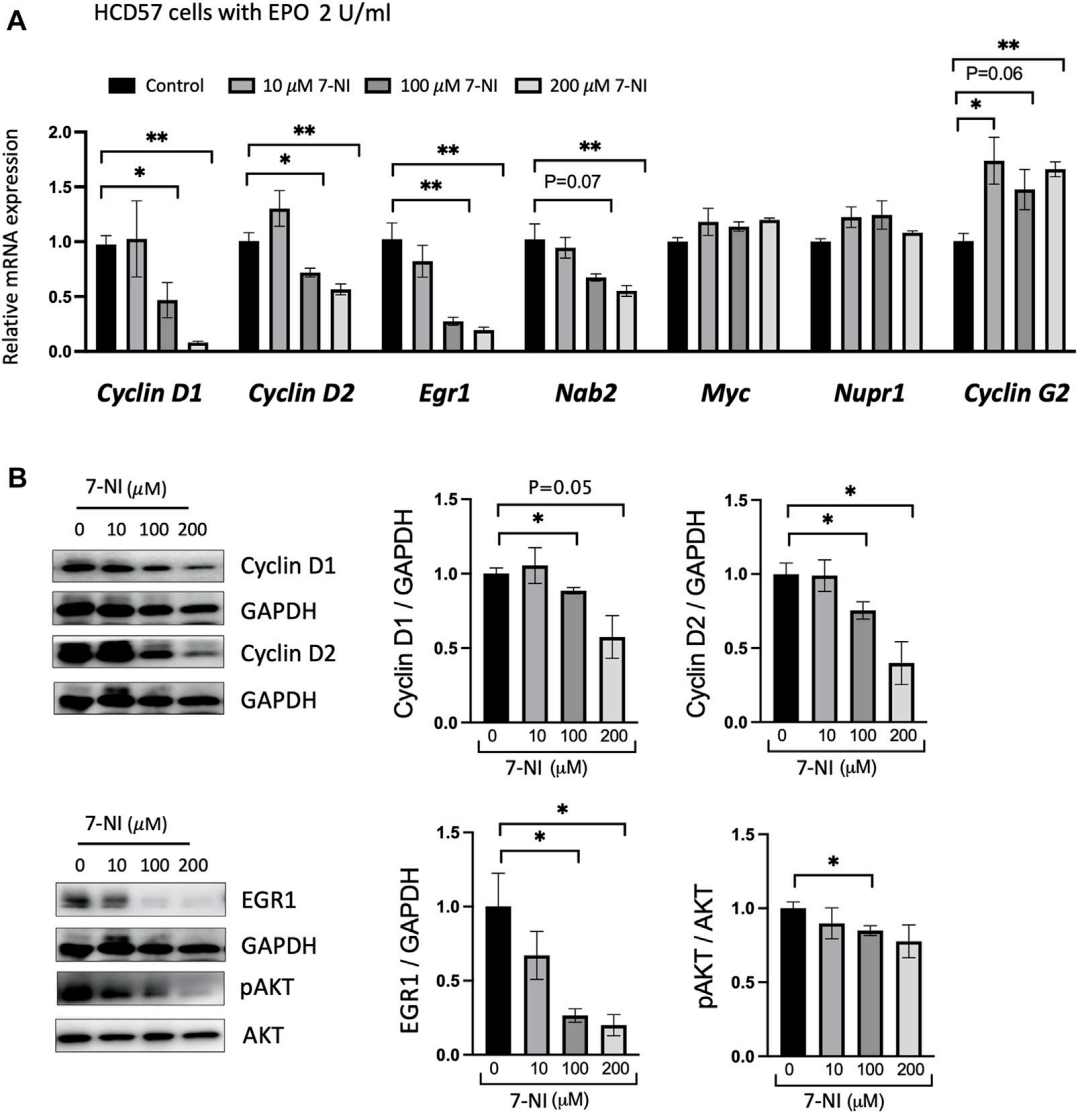
nNOS and cell cycle-associated gene expression in EPO-dependent proliferating erythroid cells. Treatment with nNOS inhibitor altered cell cycle-associated gene expression in HCD57 cells. **(A)** Gene expression was assessed by quantitative RT-PCR analysis for cell cycle-associated genes, *Cyclin D1, Cyclin D2, Egr1, Nab2, Myc, Nupr1* and *Cyclin G2* in HCD57 cells cultured with EPO (2 U/mL) and increasing concentrations of nNOS inhibitor 7-NI at 0, 10, 100 and 200 μM, and harvested at day 4 (*n* = 3/group). **(B)** Protein expression of Cyclin D1, Cyclin D2, EGR1, pAKT (Ser473), AKT and GAPDH as control was assessed by Western blotting (*n* = 3–4/group). (Indicated are ∗ *p* < 0.05, and ∗∗ *p* < 0.01).

EPO stimulated proliferating HCD57 cells were treated with increasing concentrations of 7-NI (10, 100 and 200 μM) and changes in expression of specific cell-cycle associated genes were determined. EPO has been reported to regulate cell-cycle-associated genes such as *Cyclin D2*, *Cyclin G2*, *Gspt1*, *Nupr1*, *Egr1*, *Nab2*, *Myc*, *p27*, and *Bcl6*, with increasing expression of *Cyclin D2, Nupr1, Egr1, Nab2, MYC*, and decreasing expression of Cyclin G2 (an inhibitory Cyclin) (Fang et al., 2007). Concomitant with decreased proliferation, EPO stimulated HCD57 cells treated with nNOS inhibitor 7-NI decreased *Cyclin D1*, *Cyclin D2*, *Egr1* and *Nab2* expression and increased *Cyclin G2* expression with *Myc* and *Nupr1* expression unchanged (Figure 4A). Egr1 and Nab2 have been indicated in the regulation of progression from G1 to S-phase, and Cyclin G2 expression was increased upon EPO withdrawal in primary erythroblasts and was indicated to restrict proliferation potential and inhibit cell cycle progression at S-phase in UT-7/EPO cells, an EPO dependent cell line (Fang et al., 2007). EPO inhibition of Cyclin G2 may enhance DNA replication and sustain erythroblast proliferation. These data suggest that the reduced cell proliferation by nNOS inhibition may be associated with inhibition of the G1 to S-phase transition by reduction of *Egr1* and *Nab2* or S-phase by increased Cyclin G2. Western blotting confirmed that nNOS inhibitor treatment in HCD57 cells decreased Cyclin D1, Cyclin D2, and Egr1 protein levels, and decreased pAKT activation (Figure 4B). EPO-induced differentiation of erythroid cells has been shown to be dependent on the PI3K/Akt signaling pathway (Myklebust et al., 2002; Tothova et al., 2021). In addition, EPO has been reported to induce progression of the cell cycle through upregulation of Cyclin D3, Cyclin E and Cyclin A, and the regulation of Cyclin expression is dependent on activation of PI3- and Akt-kinase pathways (Sivertsen et al., 2006). pAKT regulates the expression of *Cyclin D2*, *Cyclin G2,* and *p27* (Adlung et al., 2017). These results provide evidence that the reduced EPO dependent cell proliferation by treatment with nNOS inhibitor might be mediated by regulating cell cycle genes *via* the AKT-kinase pathway.

### 3.5 Inhibition of nNOS decreases proliferation of differentiating human primary erythroid progenitor cells and affects cell cycle related gene expression

**FIGURE 5:**
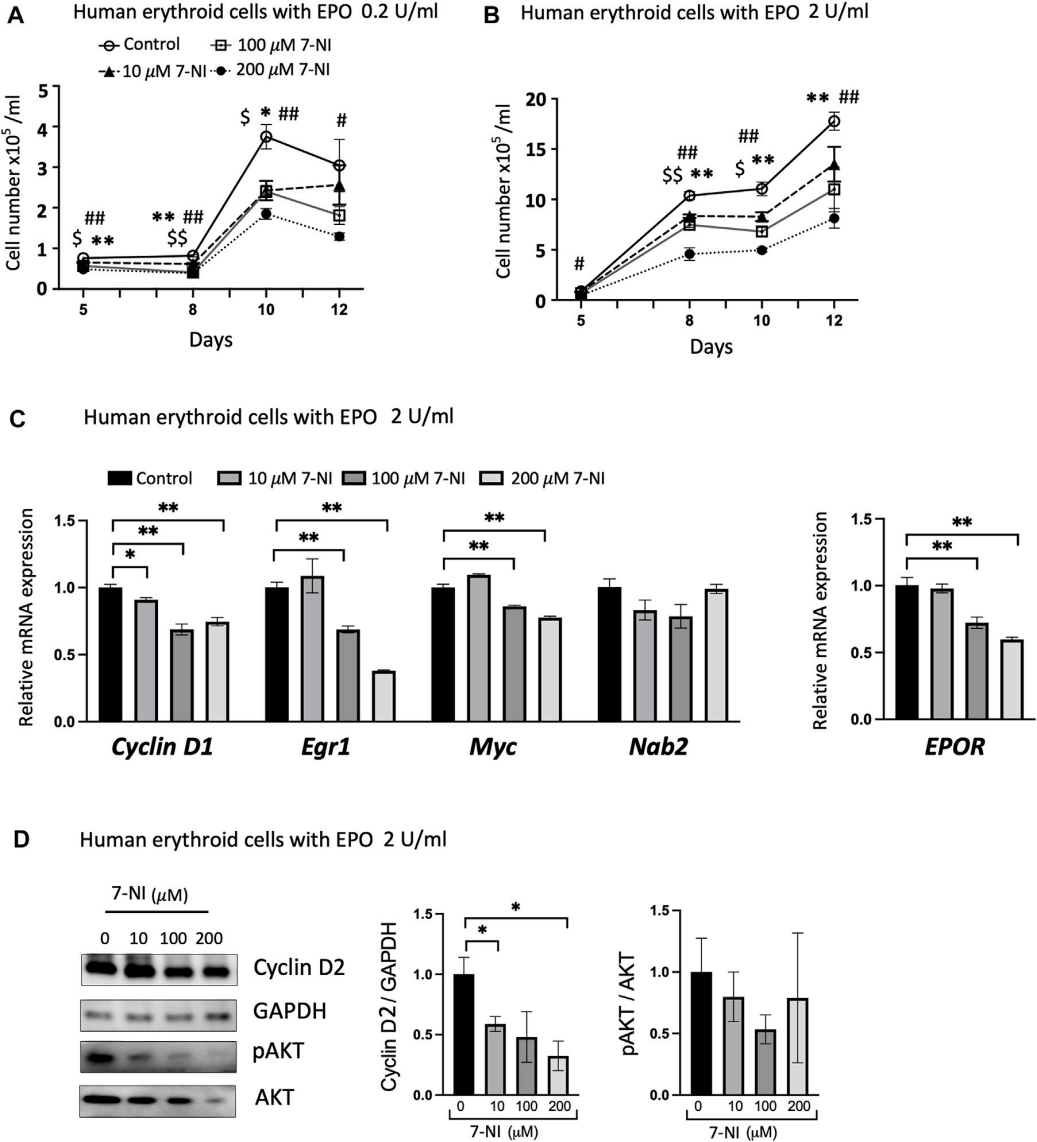
nNOS modulates proliferation of differentiating human erythroid progenitor cells. Treatment with nNOS inhibitor during EPO stimulated differentiation of primary human peripheral blood erythroid progenitor cells decreased proliferation and affected cell cycle related genes. **(A,B)** Growth curves of primary human erythroid progenitor cells stimulated with 0.2 U/mL **(A)** and 2 U/mL **(B)** EPO were treated with increasing doses of nNOS inhibitor 7-NI (0, 10, 100, 200 μM) for 12 days (*n* = 3/group; indicated are $, *, #*p* < 0.05, and $$, **, ##*p* < 0.01; $ means control vs. 10 μM 7-NI, * means control vs. 100 μM 7-NI, # means 200 μM 7-NI). **(C)** At day 10 of culture with EPO treatment at 2 U/mL, expression of cell cycle-associated genes, *Cyclin D1, Egr1, Myc* and *Nab2*, and *EPOR* was determined by quantitative RT-PCR (*n* = 3/group). **(D)** Protein expression of Cyclin D2 with GAPDH as control and pAKT (Ser473)/AKT was determined by Western blotting (*n* = 3–4/group). (Indicated are ∗ *p* < 0.05, and ∗∗ *p* < 0.01).

Primary human peripheral blood erythroid progenitor cells exhibited EPO dependent proliferation as shown for cultures treated with EPO at 0.2 U/mL (Figure 5A) and at 2 U/mL (Figure 5B), as expected (Fibach, 1998; Rogers et al., 2008). Proliferation of primary erythroid progenitor cells was sensitive to nNOS inhibition and decreased with increasing dose of 7-NI (10, 100, 200 μM; Figures 5A, B), providing further evidence that nNOS inhibition decreases EPO dependent erythroid cell proliferation as also observed with 7-NI treated proliferating HCD57 cells (Figures 3A, B). EPOR increased during EPO stimulated erythroid differentiation as reported previously (Rogers et al., 2008), but was decreased with 7-NI incubation in a dose dependent manner (Figure 5C), consistent with the decreased EPO stimulated erythroid cell proliferation (Figures 5A, B). 7-NI treatment dose-dependently modified expression of cell cycle associated genes, decreasing *Cyclin D1, Egr1*, and *Myc* expression with a tendency toward decreased *Nab2* expression (Figure 5C). The decreases in proliferation and the modification of cell cycle associated gene expression by nNOS inhibition are consistent with observations in EPO stimulated HCD57 cells treated with 7-NI (Figures 3A, B, 4A). As observed in HCD57 cells, Western blotting demonstrated that nNOS inhibition in EPO stimulated differentiating human erythroid progenitor cells decreased Cyclin D2 with a trend toward decreased AKT activation (Figure 5D) in addition to decreasing cell proliferation (Figures 5A, B).

## 4 Discussion

In non-erythroid tissue, EPO regulation of nitric oxide synthase, especially eNOS and iNOS, contributes to protection of vascular endothelium and inflammation, and nNOS is involved in EPO mediated neural protection. EPO neuroprotective activity including neural cell survival and prevention of apoptosis has been shown to be mediated *via* NO production and neural cell expression of EPOR (Chen et al., 2010). NO regulated neural cell transcription of *EPOR* and NO treatment or hypoxia induced NO increased *EPOR* reporter gene activity. Primary mouse neural cell cultures treated with NO or subjected to hypoxia induced NO showed increased number of EPOR expressing neurons that was inhibited by treatment with nNOS inhibitor. EPO stimulation of vascular endothelium in culture and animal models promotes eNOS expression and NO production, and is associated with prevention or improvement of endothelial dysfunction (Beleslin-Cokic et al., 2004; Mihov et al., 2009a; Teng et al., 2011; Serizawa et al., 2015). In rodent models of inflammation including sepsis and seizure, EPO protective activity was associated with increased eNOS, suppression of proinflammatory cytokines, and decreased iNOS expression (Contaldo et al., 2011; Kandasamy et al., 2016; Peng et al., 2020). EPO also reduced iNOS expression in inflammation during diet induced obesity (Alnaeeli et al., 2014; Lee et al., 2021). iNOS plays a critical role in development of inflammatory response in conditions such as acute lung injury, septic shock and burn injury (Nakazawa et al., 2017; Wang et al., 2020; Golden et al., 2021). Chronic inflammation and inflammatory cytokine production contributed to decreased EPO stimulated erythropoiesis resulting in anemia of chronic disease (Weiss et al., 2019; Paulson et al., 2020a). The link between stress erythropoiesis and inflammation suggests a potential role for iNOS in stress erythropoiesis (Paulson et al., 2020b). Association between iNOS and erythropoiesis is also suggested by erythroid progenitor cells from β-thal/HbE patients that exhibited increased sensitivity *in vitro* to cytokine-induced apoptosis mediated by iNOS activity (Kheansaard et al., 2011). Increased pro-inflammatory cytokines resulted in a shift in hematopoietic stem cells toward myeloid lineage commitment and reduced differentiation into erythroid and lymphoid lineages (Pietras et al., 2016). In addition to inflammatory cytokines, anemia of chronic disease has been associated with other factors such as IL-33 mediated by binding to its receptor ST2 on erythroid progenitors and high mobility group box-1 protein HMGB1 binding to its receptor on erythroid precursors, that is proposed to interfere with EPO binding to EPOR (Swann et al., 2020; Dulmovits et al., 2022).

Using EPO stimulation of erythropoiesis in WT, *eNOS−/−* and *nNOS−/−* mouse models and in corresponding bone marrow cell cultures, we provide evidence that nNOS, but not eNOS, is required for normal erythropoietic response to EPO treatment (Figure 1). EPO treatment in WT and *eNOS−/−* mice stimulated red blood cell production and increased hematocrit while hematocrit from *nNOS−/−* mice showed a markedly reduced response with EPO treatment. Furthermore, cultures of bone marrow cells isolated from WT, *eNOS−/−* and *nNOS−/−* mice suggest that nNOS is required for robust erythropoietic response of hematopoietic cells to high EPO. Erythroid colony formation assay of isolated bone marrow showed high EPO increased the number of erythroid colonies and colony size in cultures from WT and *eNOS−/−* mice. Minimal difference was evident in erythroid colony number and colony size from *nNOS−/−* mice exposed to low and high EPO. These observations suggest that nNOS is essential for sensitivity of erythropoietic response to EPO level in mice *in vivo* and in cultures of bone marrow hematopoietic cells. Transplantation of bone marrow from donor WT and *nNOS−/−* mice into immunodeficient mice demonstrated *in vivo* that nNOS contributed importantly to erythropoietic response of bone marrow hematopoietic cells to high EPO treatment (Figure 2). In erythroid cell cultures, nNOS supports proliferation in response to EPO stimulation and during erythroid differentiation (Figure 4). Treatment with NO inhibitor 7-NI showed a dose dependent decrease in cell proliferation of EPO dependent erythroid HCD57 cells and of EPO stimulated differentiating primary human peripheral blood erythroid progenitor cells. In EPO independent K562 cells, 7-NI treatment did not affect proliferation in undifferentiated erythroid cells. Decreased EPOR expression is concomitant with the decrease in EPO stimulated proliferation with nNOS inhibition in HCD57 cells and primary human erythroid progenitor cells. This is consistent with nNOS regulation of EPOR transcription in neuronal cells (Chen et al., 2010). In contrast, K562 cells that proliferate independent of EPO express very low level of EPOR (Fraser et al., 1988) (Shinjo et al., 1997). Inhibition of nNOS in EPO stimulated HCD57 cells and primary human erythroid progenitor cells decreased proliferation concomitant with decreased AKT activation and decreased expression of proteins associated with cell cycle progression such as Cyclin D1, Cyclin D2, and EGR (Figures 4, 5). During EPO stimulation of erythroid progenitor cells, AKT activation is required for erythroid differentiation and increases phosphorylation of GATA-1 and enhances GATA-1 activity to upregulate red blood cell gene expression including EPOR (Chin et al., 1995, Zhao et al., 2006). Inhibition of nNOS reduced proliferation of hemin induced erythroid differentiating K562 cells suggesting a role for nNOS in promoting erythroid differentiation independent of EPO stimulated proliferation. In addition to its role in erythropoietic response to EPO stimulation, nNOS modulates granulopoiesis and neutrophil differentiation *via* NO generation (Sadaf et al., 2021). Treatment with nNOS inhibitor 7-NI in mice abrogated granulopoiesis and decreased the numbers of bone marrow progenitor and mature neutrophils. In cultures of human hematopoietic CD34^+^ cells and K562 cells, treatment with NO donor enhanced neutrophil differentiation, and treatment with NO inhibitor in CD34^+^ cells or silencing nNOS in K562 cells reduced neutrophil differentiation. These data together with the blunted erythropoietic EPO response of *nNOS−/−* mice suggest that nNOS contributes to both erythroid and myeloid differentiation of hematopoietic stem/progenitor cells that includes EPO stimulated erythropoiesis, as well as granulopoiesis and neutrophil differentiation.

A critical role for nNOS in proliferation and tissue development in non-erythroid tissues is exemplified by bone formation in *nNOS−/−* mice that show reduced chondrocyte proliferation and bone growth (Yan et al., 2012). *nNOS−/−* mice exhibit a reduction in growth plate replicating cells with decreased Cyclin D1, slower cell cycle progression and premature cell cycle exit, thinner cortical bone and fewer trabeculae. In the nervous system, rat Schwan cells treated with nNOS inhibitor showed arrested cell cycle progression and decreased proliferating cell nuclear antigen levels (Shen et al., 2008). The proliferative activity associated with nNOS is not observed with eNOS. A major function of eNOS is regulation of vascular tone *via* NO production and of vascular endothelial growth factor induced angiogenesis (Fukumura et al., 2001; Smith et al., 2021). In a rodent model of hind limb ischemia, intramuscular gene transfer of an eNOS expression vector increased eNOS, NO, vascular endothelial growth factor and angiogenesis (Namba et al., 2003). In contrast, overexpression of eNOS directly in endothelial cell cultures inhibited endothelial cell proliferation and knockdown of eNOS increased proliferation, reduced cell cycle inhibitor p21 and increased proliferation marker Ki67, but reduced angiogenesis (Kader et al., 2000; Bu et al., 2022). Post-translational mechanisms and proper localization to intracellular compartments contribute further to nNOS and eNOS regulation. For example, NOSIP can negatively modulate NOS activity and affect endothelial cell eNOS translocation from the plasma membrane and other subcellular compartments to impair NO production and promote cell cycle regulated inactivation of eNOS and impaired NO production (Dedio et al., 2001; Schleicher et al., 2005). In neuronal cells, NOSIP influences nNOS subcellular distribution and may regulate synaptic availability and activity of NOS to protect against excessive NO production in neurons (Dreyer et al., 2004). In hematopoietic cells, NOSIP impacts on nNOS induction of neutrophil differentiation (Sadaf et al., 2021).

The range of processes linked to NO that include cell proliferation, modulation of cell cycle, angiogenesis and inflammation, appear to be differentially regulated by NO concentration that may reflect, in part, varying sensitivity to NO of specific proteins such as AKT, ERK, HIF1α, p53 and caspase (Thomas et al., 2008). Generally lower NO concentrations have been linked to survival and proliferation while higher NO concentration has been linked to cell cycle arrest and apoptosis. Furthermore, low NO concentrations can promote mitochondria respiration that is inhibited at high concentrations (Bailey et al., 2019; Dynnik et al., 2020). Here, we provide evidence that nNOS is a critical contributor to regulation of EPO dependent erythroid cell proliferation and EPOR expression, and expression of cell cycle associated genes. More detailed studies are required to determine the role of nNOS translocation during EPO stimulated erythropoiesis and of nNOS activation of the erythroid program including EPOR expression during differentiation.

## Data Availability

The original contributions presented in the study are included in the article/Supplementary Material, further inquiries can be directed to the corresponding author.

## Supplementary material

SUPPLEMENTARY FIGURE S1 Representative FACS plots for CD45.1 and CD45.2 cells from peripheral blood of Pep Boy mice with transplant from WT mice.

SUPPLEMENTARY FIGURE S2 Apoptotic cell numbers with the treatment with 200 μM 7-NI compared with PBS after 4 days of culture in HCD57 cells.

SUPPLEMENTARY FIGURE S3 Western blot of HCD57 and human erythroid cells.

SUPPLEMENTARY TABLE S1 Primer sets for quantitative real-time RT-PCR.

SUPPLEMENTARY TABLE S2 List of antibodies for Western Blotting.
