# Supplementary figures and images for: Neuronal nitric oxide synthase is required for erythropoietin stimulated erythropoiesis in mice
Source: Front Cell Dev Biol. 2023 Feb 21;11:1144110. doi: 10.3389/fcell.2023.1144110 (PMC9988911; doi:10.3389/fcell.2023.1144110)

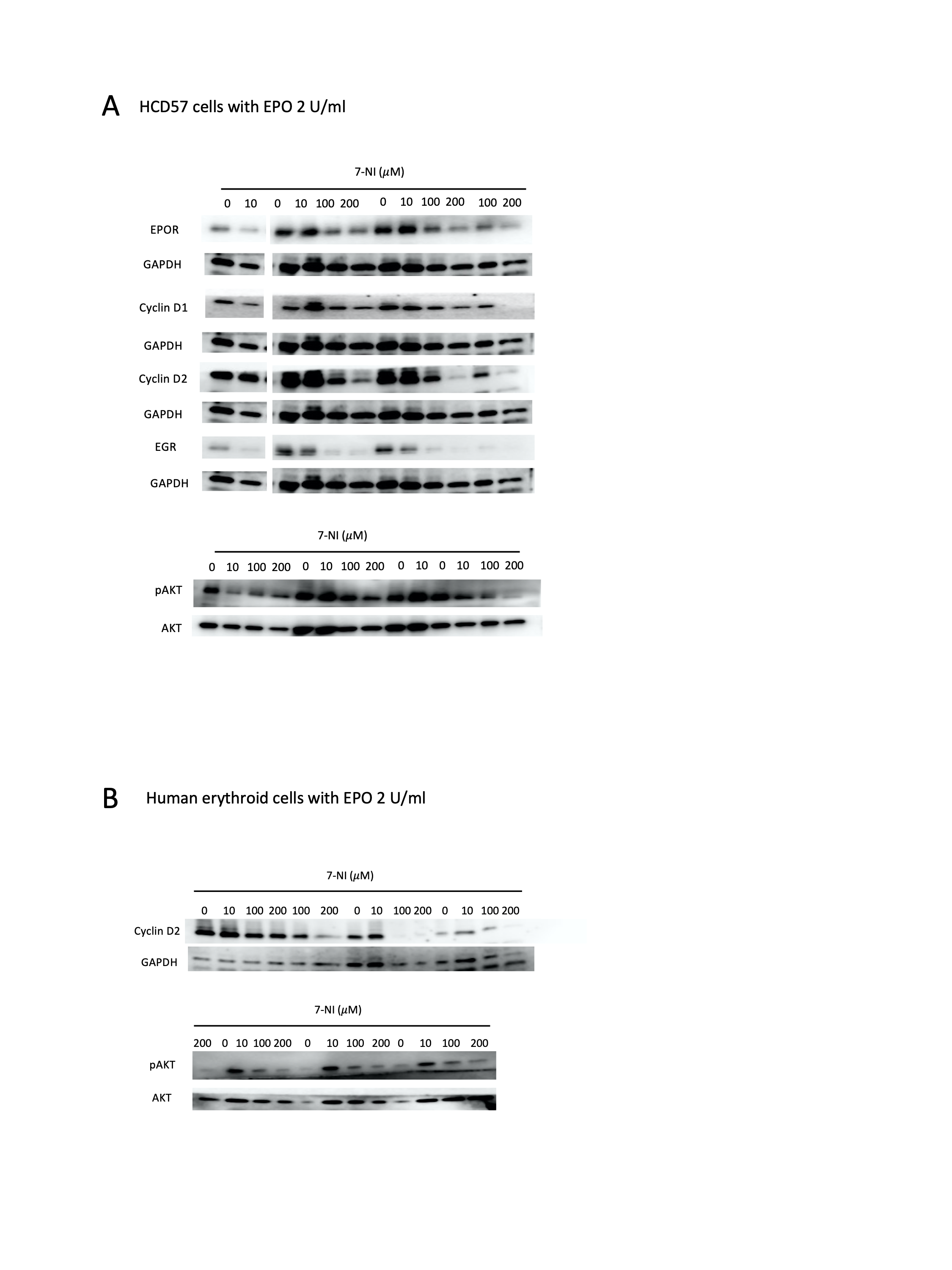

Supplement: Supplementary file 1 [file Image3.TIFF]

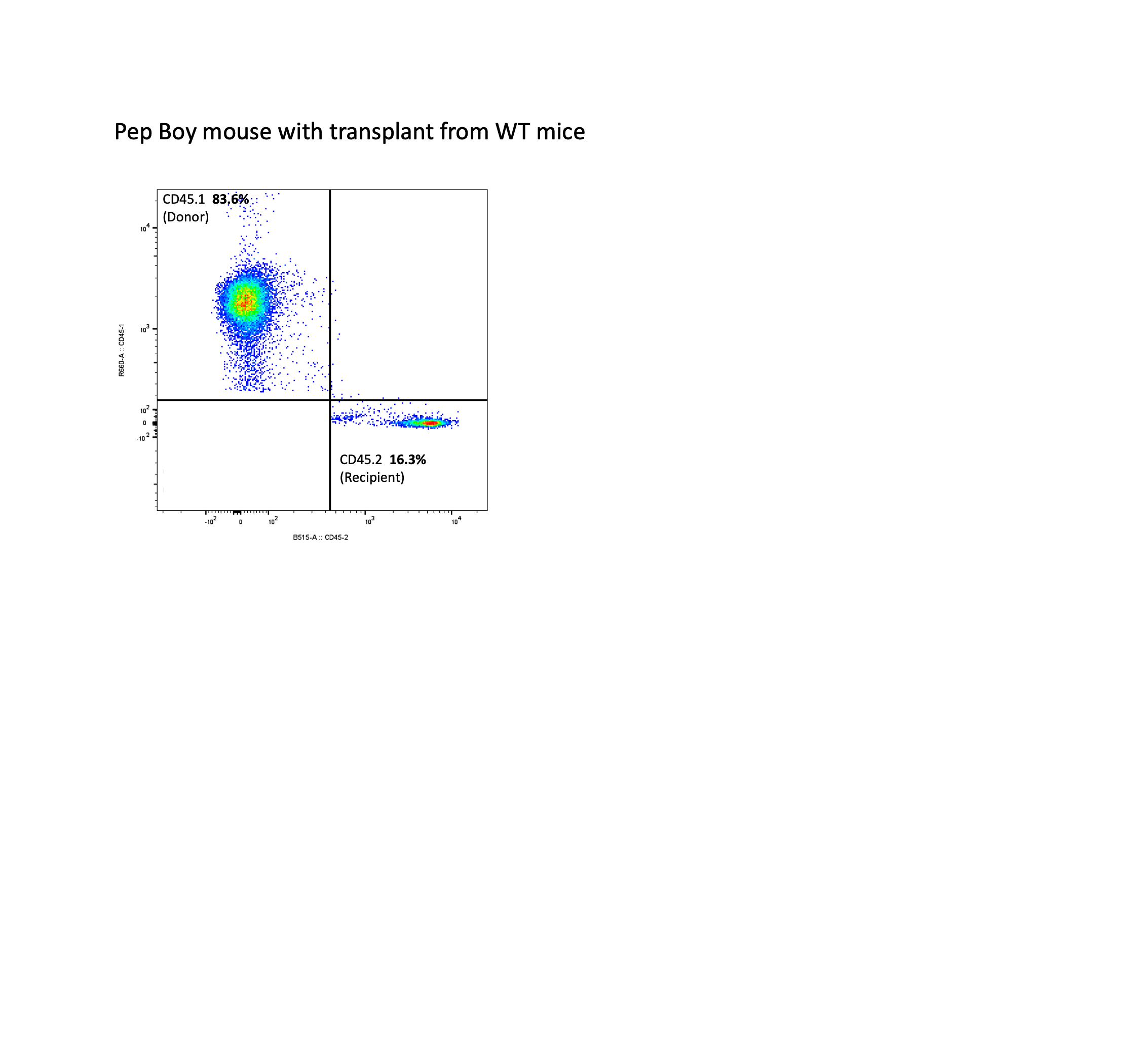

Supplement: Supplementary file 2 [file Image1.TIFF]

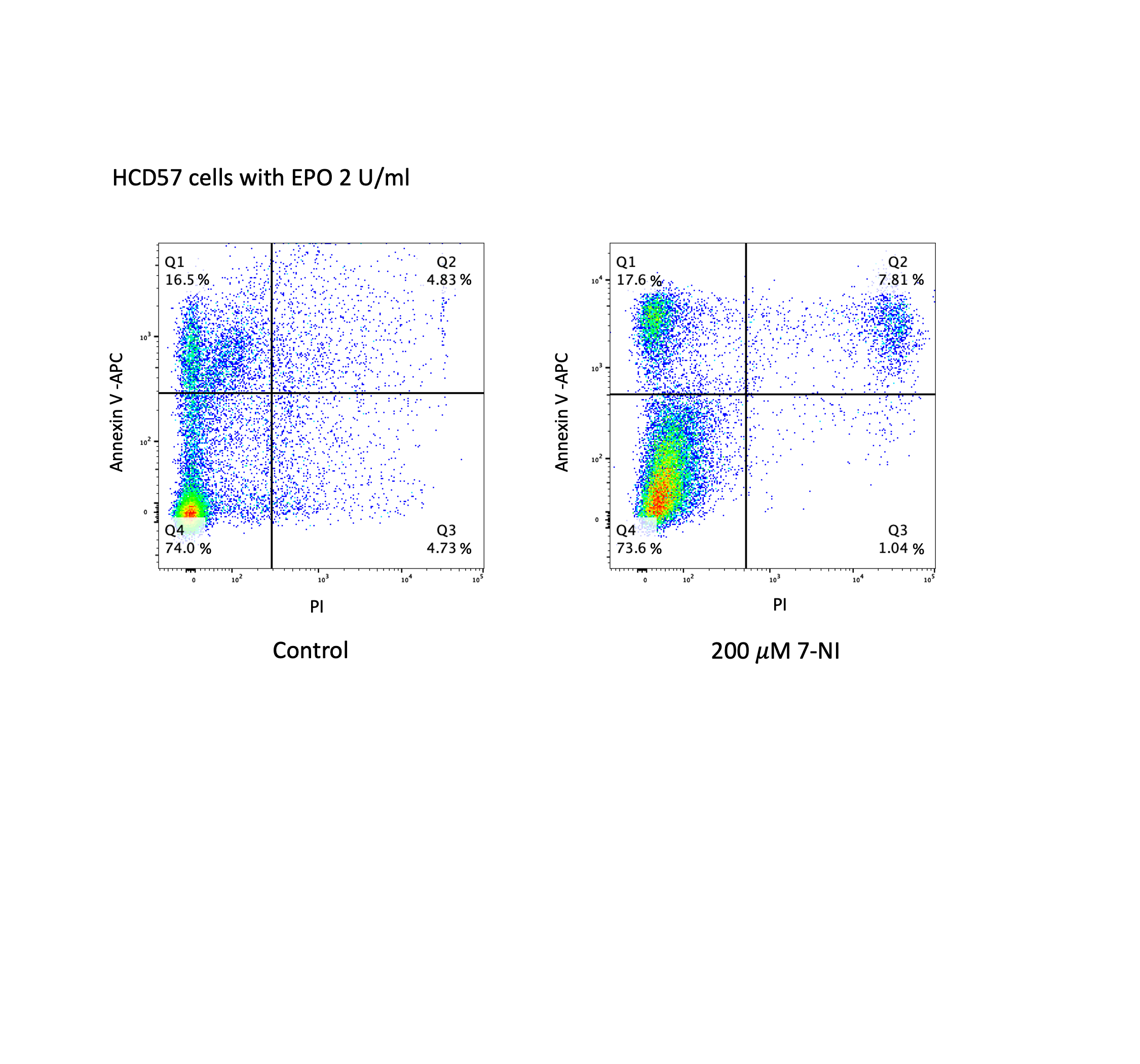

Supplement: Supplementary file 4 [file Image2.TIFF]
