# Supplementary material for: Neuronal nitric oxide synthase is required for erythropoietin stimulated erythropoiesis in mice
Source: Front Cell Dev Biol. 2023 Feb 21;11:1144110. doi: 10.3389/fcell.2023.1144110 (PMC9988911; doi:10.3389/fcell.2023.1144110)
Supplement: Supplementary file 3 [file Table1.DOCX]

| Gene | Forward | Reverse |
| --- | --- | --- |
| Mouse-Cyclin D1 | CGCAAGCATGCACAGACCTT | GCAGTCCGGGTCACACTTGA |
| Mouse-Cyclin D2 | ACACTCACGTGTGATGCCCT | ATCCGGCGTTATGCTGCTCT |
| Mouse-Cyclin G2 | TCTTGATGGAGGCTACCCCG | GCCAATGCAGGACAGGTGTT |
| Mouse-Egr1 | CAATCCTCAAGGGGAGCCGA | TGATGGGAGGCAACCGAGTC |
| Mouse-Nab2 | CTATGCACCCCCATACCGCC | CCCTCATCCATCAGTGTCTGCT |
| Mouse-Nupr1 | CAACCCTTCCCAGCAACCTC | AGCAGCTTCTCTCTTGGTCCG |
| Mouse-Myc | GCGGACACACAACGTCTTGG | CCTTGGGGGCCTTTTCGTTG |
| Mouse-EPOR | GCTCCGGGATGGACTTCA | GAGCCTGGTGCAGGCTACAT |
| Mouse-RPL13a | GCTTCTTCTTCCGATAGTGCATC | AGCCTACCAGAAAGTTTGCTTAC |
| Human-Cyclin D1 | AGATGAAGGAGACCATCCCCC | CCACTTGAGCTTGTTCACCA |
| Human-Egr1 | GCAGCACCTTCAACCCTCA | GTCTCCACCAGCACCTTCTC |
| Human-Myc | GGAGGCTATTCTGCCCATTT | GGCTGCTGGTTTTCCACTAC |
| Human-Nab2 | GGTCATGTCCAAGGCTGACG | AGGATGTGTCGGCTCCATAGC |
| Human-EPOR | GCTCCCTTTGTCTCCTGCT | CTCCCAGAAACACACCAAGTCCT |
| Human-βActin | CCTGGCACCCAGCACAAT | GCCAGTCCACACGGAGTACT |

Supplementary Table S1. Primer sets for quantitative real-time RT-PCR

Supplementary Table S2. List of antibodies for Western Blotting

| Antibodies | Concentration for Western Blotting | Catalog number | RRID | Sources |
| --- | --- | --- | --- | --- |
| CCND1 | 1:1000 | ab134175 | AB_2750906 | Abcam |
| CCND2 | 1:1000 | #3741T | AB_2070685 | Cell Signaling Technology |
| pAKT | 1:1000 | #9271 | AB_329825 | Cell Signaling Technology |
| AKT | 1:1000 | #9272 | AB_329827 | Cell Signaling Technology |
| Egr1 | 1:1000 | #4154 | AB_2097035 | Cell Signaling Technology |
| EPOR | 1:1000 | M-20 | AB_631468 | Santa Cruz Biotechnology |
| GAPDH | 1:1000 | Ab9485 | AB_307275 | Abcam |
